# Influence of the Polyvinyl Pyrrolidone Concentration on Particle Size and Dispersion of ZnS Nanoparticles Synthesized by Microwave Irradiation

**DOI:** 10.3390/ijms131012412

**Published:** 2012-09-27

**Authors:** Nayereh Soltani, Elias Saion, Maryam Erfani, Khadijeh Rezaee, Ghazaleh Bahmanrokh, Gregor P. C. Drummen, Afarin Bahrami, Mohd Zobir Hussein

**Affiliations:** 1 Department of Physics, Faculty of Science, University Putra Malaysia, 43400UPM Serdang, Selangor, Malaysia; E-Mails: elias@science.upm.edu.my (E.S.); maria_395@yahoo.com (M.E.); ghazalehbahmanrokh@yahoo.com (G.B.); 2 Department of Nuclear Engineering, Faculty of Advance Sciences and Technologies, University of Isfahan, Isfahan 81746-73441, Iran; E-Mail: rezaee.nucl@gmail.com; 3 Bionanoscience and Bio-Imaging Program, Cellular Stress and Ageing Program, Bio& Nano-Solutions, D-40472 Düsseldorf, Germany; E-Mail: gpcdrummen@bionano-solutions.de; 4 Department of Chemistry, Faculty of Science, University Putra Malaysia, 43400UPM Serdang, Selangor, Malaysia; E-Mail: mzobir@science.upm.edu.my

**Keywords:** zinc sulfide, nanoparticle, quantum dot, polyvinyl pyrrolidone, PVP, size, microwave irradiation, dispersion, bandgap, thioacetamide, sodium sulfide

## Abstract

Zinc sulfide semiconductor nanoparticles were synthesized in an aqueous solution of polyvinyl pyrrolidone via a simple microwave irradiation method. The effect of the polymer concentration and the type of sulfur source on the particle size and dispersion of the final ZnS nanoparticle product was carefully examined. Microwave heating generally occurs by two main mechanisms: dipolar polarization of water and ionic conduction of precursors. The introduction of the polymer affects the heating rate by restriction of the rotational motion of dipole molecules and immobilization of ions. Consequently, our results show that the presence of the polymer strongly affects the nucleation and growth rates of the ZnS nanoparticles and therefore determines the average particle size and the dispersion. Moreover, we found that PVP adsorbed on the surface of the ZnS nanoparticles by interaction of the C–N and C=O with the nanoparticle’s surface, thereby affording protection from agglomeration by steric hindrance. Generally, with increasing PVP concentration, mono-dispersed colloidal solutions were obtained and at the optimal PVP concentration (5%), sufficiently small size and narrow size distributions were obtained from both sodium sulfide and thioacetamide sulfur sources. Finally, the sulfur source directly influences the reaction mechanism and the final particle morphology, as well as the average size.

## 1. Introduction

Semiconductor nanostructured quantum dots have attracted much interest from virtually all scientific disciplines, including chemistry, physics, materials science, and even biology and biomedical science. This interest stems from the fact that in the nanoscale size range they show excellent and unique properties, most importantly their size tunable optical and electrical properties. The size dependence of the bandgap is one of the most important aspects of quantum confinement in semiconductors; the bandgap increases with decreasing particle size. Consequently, this size-tunability in quantum dots offers a major advantage over their bulk counterparts, which can be exploited when formulating new composite materials for a range of applications [1–3].

Zinc sulfide nanoparticles (ZnS-NPs) are important group II–IV semiconductors with a direct wide band gap energy of 3.68 eV (345 nm) [4–7] and have recently attracted significant attention because they are easy to synthesize in the required size range. Furthermore, ZnS-NPs show great potential in applications such as optoelectronic devices, light emitting displays, photocatalysis, solar cells and luminescent materials [8–10]. Since many of these applications require an appropriate average particle size and size distribution, methods that strive to meet exactly this requirement have been under intense investigation. In recent years, promising results have been achieved by performing ZnS-NP synthesis in the presence of various polymers. Surface capping of nanoparticles with polymers results in a nanoparticle product with enhanced thermal stability, and reduced reactivity and agglomeration tendency [11–13]. Moreover, the optimization of the ZnS particle size distribution, morphology, and the crystallinity is required to control their tunable intrinsic properties [4].

In recent years much effort has been put into the development of new strategies for synthesizing nanocrystals with high dispersion and size uniformity [6]. Many synthesis methods have been developed to prepare group II–VI semiconductor nanoparticles, including chemical methods [14], solid-state reactions [15], sol-gel processes [16], sonochemical preparation [17], and microwave [18,19] and gamma irradiation [20]. Among the aforementioned methods for semiconductor nanoparticle synthesis, the microwave assisted route is one of the more recent methods and is a rapidly developing area of research [3]. A major advantage of the microwave synthesis method is that it provides homogenous internal and volumetric heating at rapid rates through interaction of the dipole moment or molecular ionic with electric and magnetic fields [7]. Furthermore, the microwave synthesis method uniquely provides efficient and uniform nucleation, and subsequent particle growth without sharp thermal gradient effects [21] and thereby is capable of producing small particles with a narrow particle size distribution and high purity [3,7].

Building on previous knowledge and research, we developed a simple microwave irradiation method for the synthesis of ZnS-NPs through the reaction of zinc acetate (Zn(CH_3_COO)_2_) and thioacetamide (C_2_H_5_NS) or sodium sulfide (Na_2_S) in an aqueous solution of polyvinyl pyrrolidone (PVP). The mechanism by which PVP reacts with precursor ions is discussed in detail and the influence of the PVP concentration and the various sulfur sources on the formation of ZnS-NPs is investigated. The obtained nanocrystals were characterized by X-ray diffraction (XRD), transmission electron microscopy (TEM), Fourier transform infrared spectroscopy (FTIR) and ultraviolet/visible spectroscopy.

## 2. Results and Discussion

**Figure 1:**
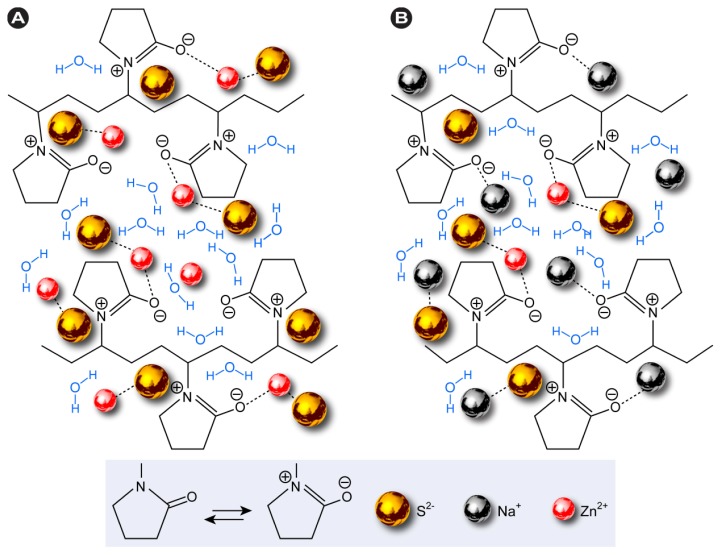
A proposed mechanism for the interactions between PVP and metal ion precursors in synthesis of ZnS-NPs using (**A**) thioacetamide and (**B**) sodium sulfide as sulfur sources.

Possible interactions between the PVP capping agent and ion precursors in the synthesis of ZnS-NPs using thioacetamide or sodium sulfide as sulfur sources are schematically shown in Figure 1. The metallic ions form stable complexes by formation of strong metallic bonds with the amide group in the polymeric chain. PVP acts as a stabilizer for the dissolved metallic salts through steric and electrostatic stabilization of the amide groups of the pyrolidine rings and the methylene groups [22,23].

**Figure 2:**
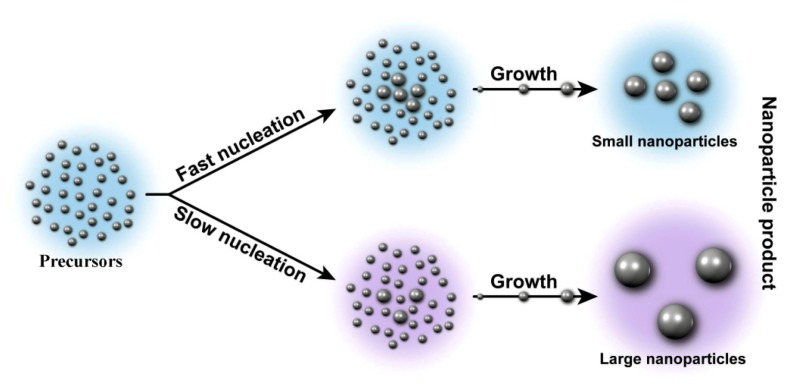
Schematic representation of the nucleation and growth of nanoparticles in the absence of Ostwald ripening.

During microwave irradiation, the PVP may be broken to a limited extent, thereby producing shorter polymer chains that reduce the capping function on the surface of the metallic ions. Since the electric component of the microwave irradiation causes heating by two main mechanisms: (i) dipolar polarization of water and (ii) ionic conduction of precursors, introduction of the polymer causes restriction of the rotational motion of dipole molecules and immobilization of ions. These factors affect the heating rate and consequently the rate of nucleation and subsequent growth of the ZnS-NPs. In a one-pot microwave-based synthesis, the relative extent of the precursor consumption between the nucleation and growth stages will dictate the final nanoparticle size, for a given amount of precursor introduced into the reaction medium. The reason for this is that particles can only grow until all of the molecular precursor is consumed (Figure 2).

Fast nucleation results in a high particle concentration and ultimately yields small particles, while a slow nucleation results in a low concentration of embryonic seeds that consume the same amount of precursor and therefore results in a population of proportionally larger particles (Figure 2) [24,25].

**Figure 3:**
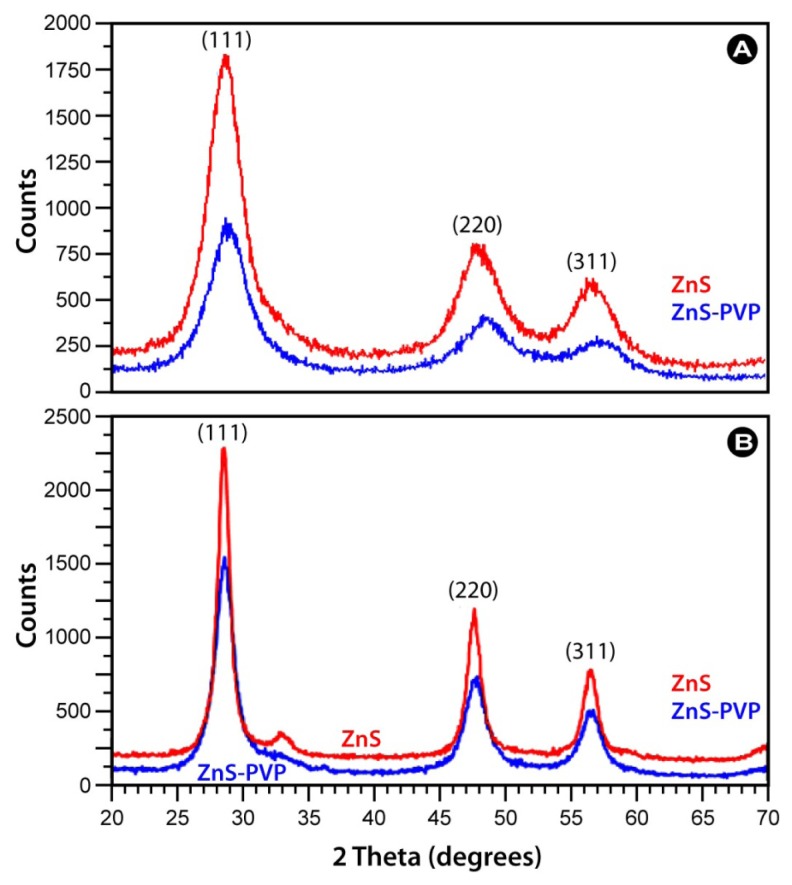
XRD pattern of the ZnS nanoparticles synthesized with (**A**) thioacetamide or (**B**) sodium sulfide as sulfur source in the absence and presence of polymer.

Figure 3 shows the XRD patterns of ZnS-NPs synthesized in the absence and presence of 3% PVP with thioacetamide or sodium sulfide as sulfur sources. The peaks observed in the XRD patterns at 2θ values of 28.6, 47.6, and 56.5, closely match the (111), (220) and (311) crystalline planes of the cubic structure of ZnS (ICDD PDF 65-0309). Furthermore, it is clearly observable that the presence of PVP attenuates the intensity of the XRD peaks compared with the absence of the polymer and all peaks shift slightly towards a higher 2*θ* value. This may be attributed to a lower degree of crystallinity and internal stress [26], which clearly supports the formation of PVP–capped ZnS-NPs.

Broad diffraction peaks are typically attributed to a smaller particle size. The crystalline size of the ZnS-NPs was calculated using the (111) reflection of the XRD patterns, based on the Scherrer Equation [3,27] according to:

(1) $$D=\frac{k\lambda }{\beta \;\text{cos }\theta }$$

where *D* is the average crystallite size, *k* is particle shape factor, λ is the X-ray wavelength (0.1542 nm), β is the angular line width of half-maximum intensity and θ is the Bragg’s angle. The estimated sizes are 4.5 nm and 9.5 nm for uncapped ZnS-NPs and 5.4 nm and 10.1 nm for PVP-capped ZnS-NPs prepared with thioacetamide and sodium sulfide respectively.

**Figure 4:**
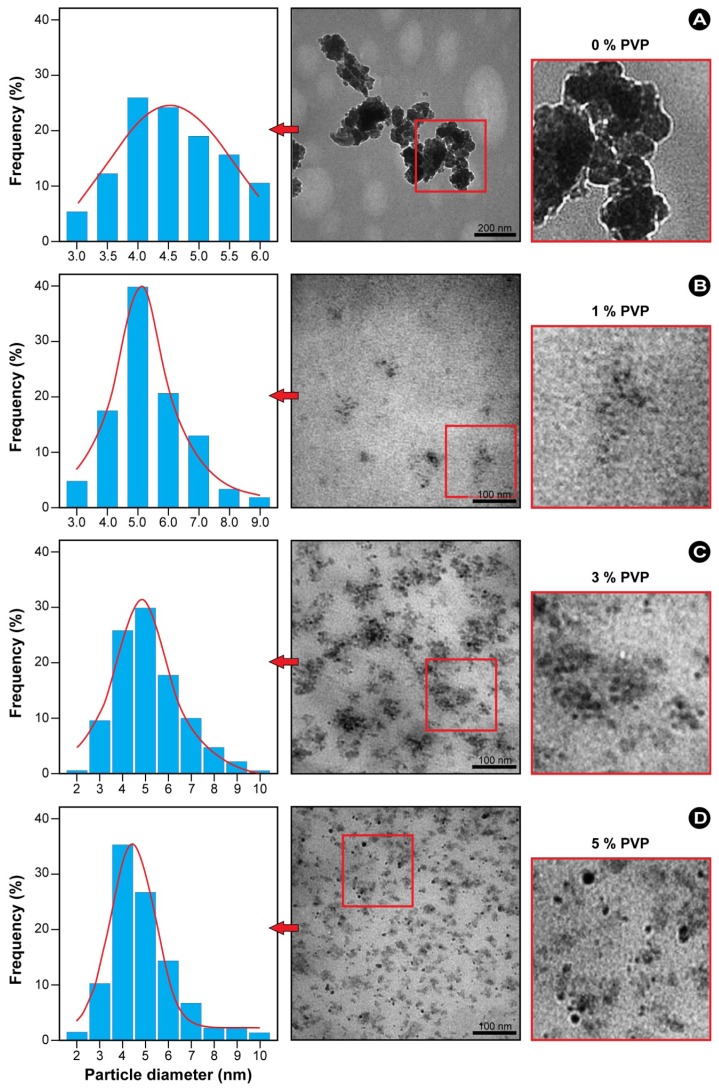
TEM images and particle size distribution of PVP-capped ZnS synthesized using various concentrations of PVP and thioacetamide as a sulfur source.

To further investigate the role of PVP in the synthesis of ZnS-NPs, experiments in which the PVP concentration was adjusted from 0% to 10% (weight ratio of PVP to water) during the synthesis were performed. The results show that both the presence of PVP and the PVP concentration significantly affect the size distributions and dispersion of the resulting nanoparticles. Figure 4 shows the TEM images and corresponding size distribution histograms of ZnS-NPs synthesized using thioacetamide at different concentrations of polymer solution. Morphological evaluation of the TEM images in Figure 4B–D shows that the ZnS-NPs are generally spherical in shape and have relatively narrow size distributions. In the absence of PVP (Figure 4A), the CH_3_COO^−^ anions bound to the surface of the ZnS-NPs restrict the particle growth. Consequently, nanoparticles with a small average size (3–6 nm) are produced, whilst the hydrogen bonds between the CH_3_COO^−^ anions promote aggregation [18], which is clearly noticeable in the TEM image in Figure 4A. Conversely, PVP-capped ZnS-NPs approximate monodispersity and have mostly larger sizes in the range from 2 to 10 nm. At low PVP concentration, some agglomerated particles can be observed (Figure 4B). However, when the PVP concentration is increased to a maximum of 5%, well isolated and dispersed nanoparticles are obtained. Furthermore, both the particle size distribution and the average particle diameter are reduced (to ~5.1 nm), as deduced from the width of the Gaussian fit at half maximum (Figure 4).

After introducing PVP into the reaction mixture, Zn^2+^ ions form a complex with PVP, which results in particle capping upon nucleation. Since the complex compound Zn(PVP)^2+^ formed stabilizes the Zn^2+^ ions, the reaction is restrained and the subsequent slower nucleation process results in the production of larger particles. Furthermore, with the increase in the PVP concentration from 1 to 5%, more Zn^2+^ ions are complexed to PVP and the growth of the particles is increasingly controlled by the formation of passivation layers on the particles’ surface. It is worth noting that further increasing the PVP concentration beyond 5% does not result in the production of smaller particles, because the reaction system becomes so viscous, and it is challenging to collect the desired ZnS-NPs.

**Figure 5:**
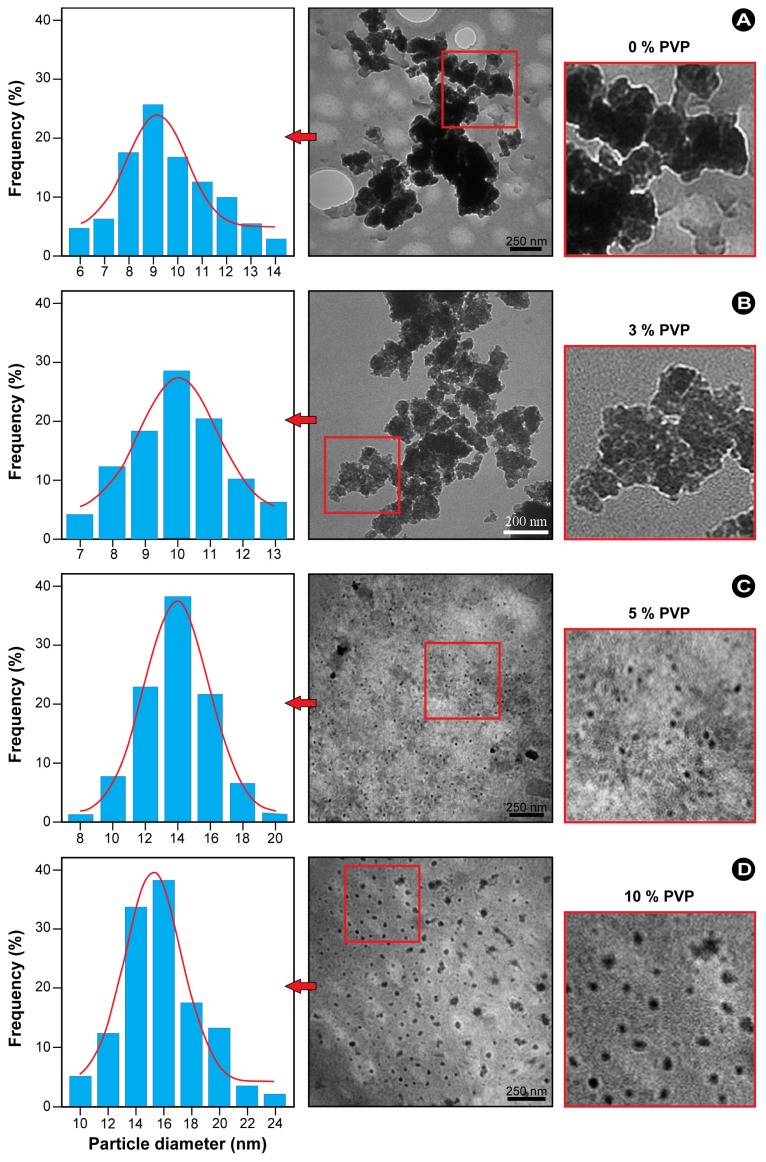
TEM images and particle size distribution of PVP-capped ZnS synthesized using various concentrations of PVP and sodium sulfide as a sulfur source.

Similar experiments using an alternate sulfur source, *i.e.*, sodium sulfide, were performed to determine the effect of the sulfur source on the final nanoparticles product. As shown in Figure 5, ZnS-NPs obtained in either the absence or at low PVP concentration (3%) show a significant degree of agglomeration and have average sizes of ~9.0 and 9.2 nm, respectively. However, when increasing the PVP concentration from 5% to 10%, monodispersed particles were obtained at the expense of a noticeably increased particle size (Figure 5C,D). The determined average sizes of these well dispersed ZnS-NPs are ~10.3 and 15.8 nm for 5% and 10% PVP, respectively.

Overall, the effect of the PVP concentration on the particle size and size distribution was more pronounced in our experiments when using sodium sulfide compared to thioacetamide. Consequently, our expectations that the sulfur source might considerably affect the reaction process and the final nanoparticles product were effectively met. When using sodium sulfide as a sulfur source, both metal ions Zn^2+^ and Na^+^ are coordinated with PVP upon introduction and the complex compounds of Zn (PVP)^2+^ and Na (PVP)^+^ are formed in which the Zn^2+^ and Na^+^ ions are stabilized. The stabilization of Zn^2+^ restrains the reaction, but the stabilization of Na^+^ actually promotes the reaction. In view of the fact that the number of Na^+^ ions is higher than the Zn^2+^ ions, the latter stabilizing effect may be stronger than the former one. Whereas monodispersed particles are preferentially formed when the speed of nucleation is much higher than the speed of particle growth [28], at low PVP concentration, the polymer cannot completely cover the Zn^2+^ ions, and consequently agglomerated particles are produced. Conversely, with increasing PVP concentration, more ions are covered by the polymer, which slows the reaction process down; as a result, the size of the nanoparticles increases and monodispersed particles are obtained. The optimized PVP concentration in the reaction medium was found to be about 5%. This concentration provided the conditions required to fabricate well dispersed ZnS-NPs, whilst obtaining a nanoparticle population with a sufficiently small average size and narrow size distribution.

In order to further study and attempt to unravel the underlying adsorption mechanism of PVP onto the nanoparticle surface, FTIR analysis of PVP-capped ZnS-NPs was carried out and the obtained data was compared with the FTIR spectrum PVP alone.

**Figure 6:**
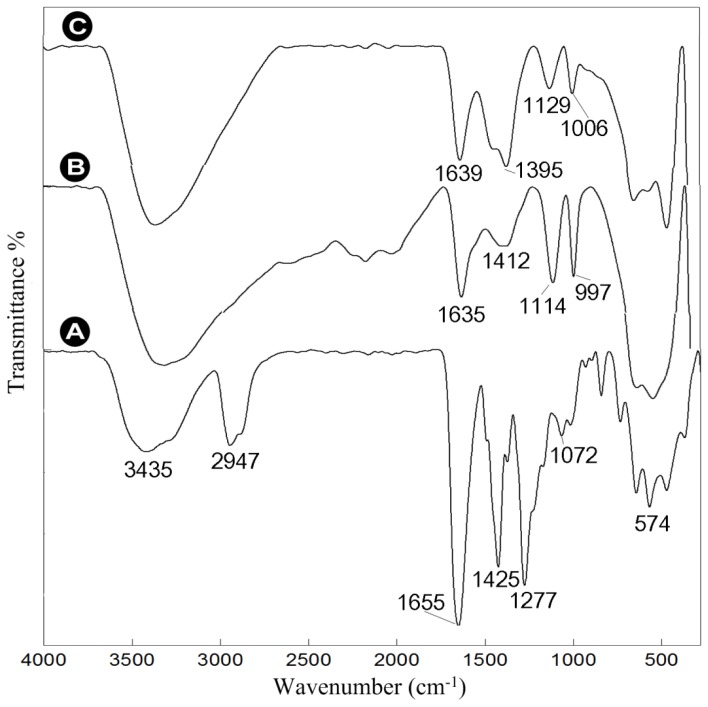
FTIR spectra of (**A**) PVP; (**B**) 5% PVP-capped ZnS-NPs, and (**C**) 3% PVP-capped ZnS-NPs, synthesized using thioacetamide as a sulfur source.

When comparing the FTIR spectra of pure PVP and PVP-capped ZnS-NPs in Figure 6, a prominent absorption peak may be observed at 1655 cm^−1^ (Figure 6A), which represents the functional C=O unit in PVP. This peak shifts to 1635 cm^−1^ (Figure 6B) and 1639 cm^−1^ (Figure 6C) for a polymer concentration of 5 and 3%, respectively. Such a decrease in wave number for the C=O stretch may be attributed to bond weakening as a result of backbonding via partial lone pair electron donation from the oxygen in PVP with surface Zn atoms, which eventually passivates the ZnS-NP surface. Moreover, the peaks at 1277 cm^−1^ and 1072 cm^−1^ (Figure 6A) may be assigned to the C–N bond stretching mode, and shift to 1114 and 997 cm^−1^ for a polymer concentration of 5% (Figure 6B) and 1129 and 1006 cm^−1^ for polymer concentration 3% (Figure 6C). Furthermore, note that the C–H peak at 1425 cm^−1^ is significantly weakened. The peak shifting towards lower wave numbers that correspond to C-N bonds is likely due to chemical coordination of ZnS-NPs with these bonds. In addition, the peak shifting of the C=O and C–N bonds becomes more pronounced with increasing PVP concentration from 3 to 5%, which corresponds to a final product of well or better dispersed nanoparticles.

**Figure 7:**
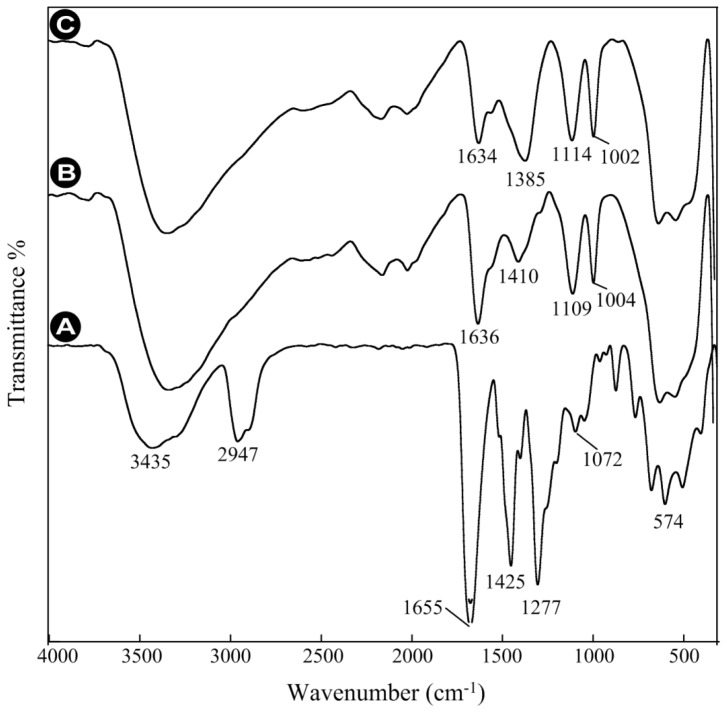
FTIR spectra of (**A**) PVP; (**B**) 10% PVP-capped ZnS-NPs, and (**C**) 5% PVP-capped ZnS-NPs, synthesized using sodium sulfide as a sulfur source.

Similarly, the nanoparticle product using sodium sulfide as a sulfur source was evaluated and the results of the FTIR measurements are presented in Figure 7. Figure 7A again shows the reference spectrum of pure PVP with the characteristic C=O absorption peak at 1655 cm^−1^, which shifts to smaller wave numbers, *i.e.*, 1636 cm^−1^ (Figure 7B) and 1634 cm^−1^ (Figure 7C), after capping with PVP.

The C-N peaks at 1277 cm^−1^ and 1072 cm^−1^ (Figure 7A) equally shift to 1109 and 1004 cm^−1^ for 10% PVP (Figure 7B) and 1114 and 1002 cm^−1^ for 5% (Figure 7C). The characteristic changes in the optical behavior of PVP indicates that the ZnS-NPs are conjugated via the N and O atoms in C=O and C–N (Figure 1). It should be noted that with increasing polymer concentration, the extent of the C–N peak shift decreases. This is due to the steric effect exerted by pyrrolidone that is more prominent with increasing polymer concentration and influences the coordination between N and the ZnS-NP surface. Consequently, ZnS-NPs produced at 5% polymer concentrations were better protected, which resulted in smaller sized particles.

**Figure 8:**
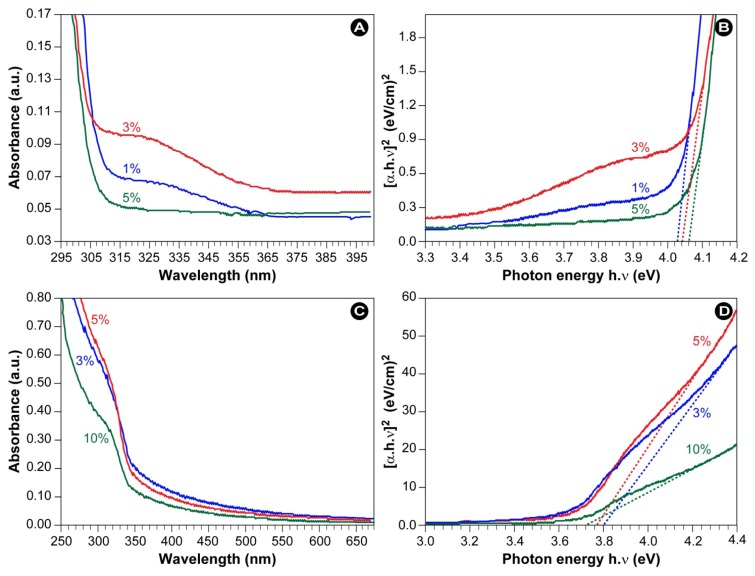
(**A**) UV–*vis* spectra and (**B**) Tauc plot of PVP-capped ZnS-NPs synthesized with thioacetamide as a sulfur source. (**C**) UV–*vis* spectra and (**D**) Tauc plot of PVP-capped ZnS-NPs synthesized with sodium sulfide as a sulfur source.

To determine the size and nature of the bandgap, UV-vis spectra were recorded, as presented in Figure 8. The samples show a strong absorption below 300 nm and a blue shift of the absorption edge compared to bulk ZnS (onset is at 340 nm [7]), which can be attributed to the quantum confinement effect.

The energy of the band gap (*E*_g_) can be calculated from the UV–*vis* spectra via a Tauc plot of (*α h v*)^2^ *versus* (*hv*) and extrapolation of the linear part of the curve to the energy axis according to [13,29–31]:

(2) $$\alpha \;h\;\nu =B{\left(h\;\nu -E_{g}\right)}^{\frac{1}{2}}$$

where α is the absorption coefficient, *hv* is the photon energy, *E_g_* is the direct band gap energy, and B is a constant. Figures 8B and D show the Tauc plots of PVP-capped ZnS-NPs. The optical band gaps were determined from the extrapolation, as indicated by the dotted lines in Figure 8, and the results are listed in Table 1. It can clearly that PVP-capped ZnS samples show an increase in the band gap energy by more than 0.05 compared to the bulk.

From the band gap values, the particle sizes were calculated using the Brus equation [13,29,30] according to:

(3) $$\Delta E=\frac{\hbar^{2}\pi^{2}}{2r^{2}}\left(\frac{1}{m_{e}^{*}}+\frac{1}{m_{h}^{*}}\right)-\frac{1.8e^{2}}{4\;\pi \;{ɛ}_{0}\;{ɛ}_{r}\;r}$$

where Δ*E* is the blue shift of the band gap, *m_e_* * and *m_h_* * are the effective mass of the electron and hole, *r* is the particle radius, *ɛ_r_* is the dielectric constant and *ɛ*_o_ is the permittivity of free space. The first term mathematically describes the confinement effect, whilst the second term is the Coulomb term.

In a strong confinement, as in the present case, the second term is small and may be neglected [2]. From Equation 3, the bandgap and the particle sizes were calculated (see Table 1). It can be seen from the table that the particle sizes are in fair agreement with those determined from the TEM images (Figures 4 and 5).

Finally, to ascertain the stability and the shelf-life of the PVP-capped ZnS-NPs, UV-vis spectra of the various samples were re-recorded after one month storage under normal conditions (ambient temperature in the dark). Stability measurements show that PVP-capped ZnS-NPs remain stabile after prolonged storage, with no significant changes in the absorption spectra. In contrast, particles produced in aqueous solution only reach moderate stability and deteriorate over time to form significantly instable colloidal solutions, which was confirmed through TEM evaluation of stored solutions. In general, the results of our stability measurements show that a good measure of colloidal stability is present and retained.

## 3. Experimental Section

The starting materials for the synthesis of ZnS nanoparticles were zinc acetate, poly-*N*-vinyl-2-pyrrolidone (PVP10), thioacetamide, and sodium sulfide. All chemicals were acquired from R&M Chemicals (Edmonton, AB, USA) or Sigma-Aldrich (Kuala Lumpur, Malaysia) and were analytical grade products, which were used without further purification.

In a typical procedure, the appropriate amount of PVP (1 g) was dissolved in 10 mL distilled water. Subsequently, 5 mM zinc acetate was added to the PVP solution under stirring (500 rpm) and the resulting solution was homogenized for 30 min. Next, 10 mL of aqueous sulfur source solution was added and stirred until a well-dissolved solution was obtained. The concentration of the sulfur sources was slightly higher than the zinc source in order to ensure that the reaction would proceed to completion. The final solutions of different sulfur sources were put in a high power microwave oven (1100 W) using a pulse regime of 20% power for 20 min. The precipitates were centrifuged (3500 rpm, 5 min) and washed several times with distilled water and ethanol. The white products were dried in air at 60 °C for 24 h in a controlled environment. To monitor the effect of the PVP concentration, adjustment of the PVP to water weight ratio from 0 to 10% were prepared during synthesis.

The products were characterized by X-ray powder diffraction (XRD) at a scanning rate of 5°/min in the 2*θ* range 20–70° using a Philips X-ray diffractometer (N.V. Philips Analytical X-ray, Almelo, the Netherlands) with Cu Kα radiation (λ = 0.1542 nm). The particle size and size distribution were determined with a Hitachi H-7100 Transmission Electron Microscope ([TEM]; Hitachi, Chula Vista, CA, USA) operating at an accelerating voltage of 100 kV. The average size and size distribution of the nanoparticles were evaluated with UTHSCA Image Tool from at least 200 nanoparticles for each sample. The chemical interaction of PVP with the ZnS nanoparticles was investigated using a Perkin-Elmer model 1650 Fourier Transform Infrared (FTIR) spectroscope (Perkin-Elmer, Waltham, MA, USA). The optical properties of the ZnS nanoparticles were characterized using UV–vis spectroscopy on a Shimadzu UV–1650PC spectrophotometer (Shimadzu, Columbia, MD, USA).

## 4. Conclusions

Well-dispersed ZnS-NPs with narrow size distributions were prepared in a polyvinyl pyrrolidone polymeric solution via a simple, rapid and energy efficient microwave synthesis method. Since the majority of atoms in a nanoparticle are located on its surface, the modification of the nanoparticle’s surface has been recognized as the prime method to tailor nanomaterials for particular applications, with a high measure of control over the final product. Differences in the ZnS-NP size and dispersion were found to be polymer concentration dependant, as clearly visible upon TEM microscopic evaluation. Therefore, capping with a polymer, such as PVP, occurs via conjugation between PVP and the ZnS-NPs and not only offers enhanced colloidal stability, but also some measure of control over the size and the size distribution. The conjugation seems to occur via C–N and C=O groups, as confirmed via FTIR analysis. After introducing the polymer, both resonance C–N and C=O peaks in the FTIR spectra changed noticeably, which is indicatory for the coordination of ZnS-NPs with the N and O atoms of PVP. The optimal PVP concentration was determined to be ~5%, which resulted in a final product with an improved dispersion and relatively small size and size distribution. The average particle sizes and estimated optical band gaps of PVP-capped ZnS-NPs synthesized under optimal conditions are 5.1 nm and 4.07 eV for thioacetamide and 15.8 nm and 3.72 eV for sodium sulfide as sulfur sources. These results also indicate the significance of the sulfur source and thus the reaction mechanism on the final product.

**Figure 9:**
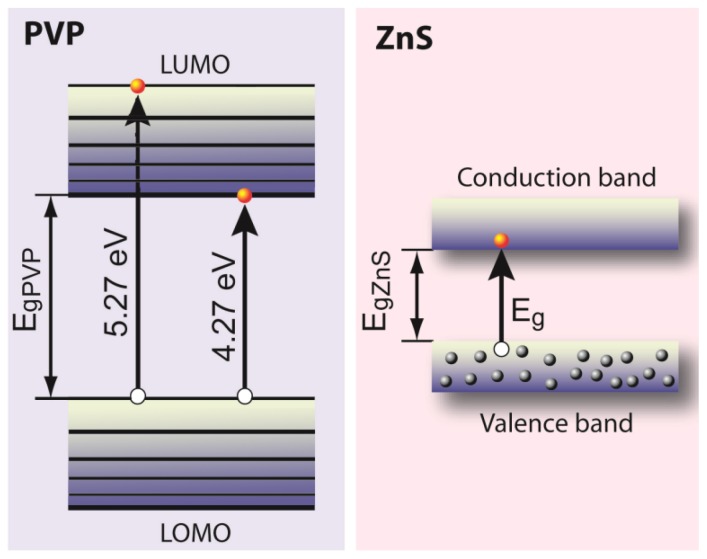
Schematic illustration of the energy bands in PVP-capped ZnS nanoparticles.

The potential of capping ZnS-NPs is amongst others illustrated by the fact that PVP-capped ZnS-NPs exhibit enhanced photoactivity and photocatalytic performance compared to bare ZnS nanoparticles. Interaction of PVP with the metal ions located at the nanoparticle’s surface can give rise to overlap of the molecular orbitals of PVP with the atomic orbitals of the metal ions. These interactions can cause both (i) alteration of the tautomeric form of surface adsorbed organic molecules due to change in the effective charge on N and C=O and (ii) transfer of the excited state energy from the molecular orbital of PVP to ZnS nanocrystals [8]. Since the PVP related energy bands (*E*_g_~4.27 eV) are very close to the ZnS-NP valence and conduction bands (Figure 9), the process of energy transfer between the surface adsorbed PVP molecules and nanoparticles can take place efficiently.

In conclusion, the present work presents an easy, inexpensive and effective way to produce well-dispersed, small, and stabile nanoparticles with a polymer coating that allows easy further functionalization and affords potentially controllable physical, especially optical properties.

## Figures and Tables

**Table 1:** Optical band gap, blue shift and particle size of PVP-capped ZnS nanoparticles.

Samples	Sulfur source	Band gap (eV)	Blue shift (eV)	Particle Size (nm) from	

				UV	TEM
ZnS/PVP (1%)	TAA^1^	4.03	0.35	5.2	5.3
ZnS/PVP (3%)	TAA	4.05	0.37	5.1	5.2
ZnS/PVP (5%)	TAA	4.07	0.39	5.0	5.1
ZnS/PVP (3%)	Na2S	3.80	0.12	8.9	9.2
ZnS/PVP (5%)	Na2S	3.77	0.09	10.2	10.3
ZnS/PVP (10%)	Na_2_S	3.72	0.04	15.5	15.8

1 TAA = thioacetamidev.
